# Doctors in Chinese public hospitals: demonstration of their professional identities

**DOI:** 10.1186/s12909-020-02339-3

**Published:** 2020-12-10

**Authors:** Zhanming Liang, Min Xu, Guowei Liu, Yongli Zhou, Peter F Howard

**Affiliations:** 1 The Second Affiliated Hospital of Shandong First Medical University, Tai’an, Shandong China; 2 La Trobe University, Bundoora, Vic Australia; 3 Shandong University of Traditional Chinese Medicine, Jinan, 250355 China

**Keywords:** Chinese public hospitals, Doctor – patient relationships, Professional identity

## Abstract

**Background:**

An increase in the number of medical disputes and violence against doctors indicates a lack of trust in the medical profession by society in Chinese public hospitals. Empirical evidence confirms that one cause is the lack of professional identity demonstrated by doctors. Medical professionals are required to maintain high standards of competence and moral responsibility, and demonstrate qualities such as respect, compassion, integrity, responsiveness to needs, and commitment to sound ethical practice in order to maintain professional privilege. These principles and appropriate professional conduct are the foundation of the professional identity of the medical profession.

**Methods:**

A quantitative approach was adopted by distributing paper-based questionnaires to doctors and patients in two hospitals (Level III and Level II) in Jinan, Shandong province, China.

**Findings:**

In total, 614 doctors and 1184 inpatients on discharge from the surgical and internal medicine units of the two hospitals participated in the survey yielding 90% response rates. The study confirmed the variation amongst doctors in demonstrating their professionalism in terms of respecting patients’ views and preferences when determining diagnostic procedures and treatment plans, and when making ethical decisions. Although 90% patients indicated that they showed respects to doctors, close to 20% of the doctors disagreed that they received high respect from patients. About 12% of doctors prescribed unnecessary diagnostic procedures to patient for the purpose of generating profit and more than 20% of patients indicated that they gave gifts to doctors in order to receive better treatment.

**Conclusions:**

Although about 80% of doctors demonstrated certain aspects of professionalism required by practitioners, the inconsistency across the medical workforce may exacerbate tense doctor-patient relationships. A review of medical curricula and focus of the internship program is required in order to assist medical graduates with forming required professional identity in order to improve patient satisfaction and better clinical outcomes. To be effective, a more systematic approach is recommended.

**Supplementary Information:**

The online version contains supplementary material available at 10.1186/s12909-020-02339-3.

## Background

Medical disputes and violence against doctors have been the centre of attention in the hospital system in China over the past one and a half decades – a crisis of the Chinese healthcare system [1]. The rapid increase of medical disputes coincided with the major healthcare reforms in China since the outbreak of Severe Acute Respiratory Syndrome (SARS) in 2003, which shifted the operation of the health system from a market-oriented to public welfare-oriented system [2]. Alarmingly, an average of 22.9% annual increase in the number of medical disputes were recorded between 2002 and 2013 in China [3]. Although the introduction of the Tort Liability Law in 2010 has had some impact on the growth, a total number of 115,000 cases were still reported in 2014. A survey of a representative sample of 326 Chinse hospital nationwide conducted by the Chinese Hospital Association in 2016 indicated that 98.4% of these hospitals had medical disputes in 2015 and spent about 5.9% of total revenue on compensation as a result [2]. Of all the medical disputes in China, 80% of them were not resolved within a legal framework but via either ‘under-the-table’ arrangements. Few ended by escalation to violence targeting doctors [4] because it was the quickest and least expensive way for patients and families to obtain large ‘pay outs’ and hospitals avoided the risk of damaging their reputation and social stability [5]. More than 70% of malpractice claims that went to trial eventually resulted in monetary compensation in China [6]. In other countries, compensation rate for malpractice claims varied in different studies. The compensation rate various across different studies in different countries. For example, few studies in the USA confirmed that 25 to 65% of malpractice claims resulted in monetary compensation [6], and a study conducted in a large region in Spain confirmed a slightly lower rate of 25% [7]. A systematic literature on malpractice claim at primary care conducted by Wallace et al. (2013) found that 31.9% of the claims made between 1985 and 2008 resulted in payment in the US, the payout rate on paediatric claims in a study in France was as high as 72% [8]. The review also found that almost half of the malpractice claims in the UK are either discontinued or successfully defended making a compensation rate much lower than 50% [8] .

Empirical evidence internationally and from China indicate that the erosion of trust in the medical profession, sensational media coverage, poor communications and attitudes including de-valuing patients’ views by medical professionals, and poor practice by staff are the major reasons behind the disputes [9, 10]. Good communications and interpersonal skills are tools that help doctors to gain trust from patients and in improving patient satisfaction and quality of patient care [11]. However, heavy workloads may have prevented adequate and effective communication between doctors and patients in Chinese public hospitals [5] and the volume-based incentive funding schemes of the Chinese public hospital system has encouraged revenue generation behaviours amongst doctors which has further damaged the trust and respect from patients and the public [2]. Moreover, the pressure of medical disputes and avoidance of malpractice claims has created tense relationships between doctors and patients with possible severe consequences of disputes [5, 6]. The unclear legal framework and procedures have turned doctors’ patterns of behaviour into a defensive mode which can be described by the emerging trend of ‘Defensive Medicine’ [12, 13]. Defensive medicine includes behaviours such as over-prescribing medical procedures, clinical tests and unnecessary medication, and avoiding risky patients and procedures makes no contribution to the quality of care but has added pressures onto the overall health care budget [14]. This has ultimately hindered the efforts and benefits of the health system reform in China.

The medical profession earns the public and patients’ trust and their privilege, independence and occupation autonomy not only from medical expertise and clinical effectiveness, but also from the social contract between the medical profession and the society [15]. To maintain such privileges, in addition to clinical effectiveness, medical professionals are also required to maintain high standards of competence and moral responsibility, and demonstrate respect, compassion, integrity, responsiveness to needs, commitment to sound ethical practice and sensitivity to culture, age, gender and disability which are viewed as core elements of professionalism to be demonstrated [16]. The basis of the public’s trust is the confidence that doctors will put their patients’ welfare ahead of all other considerations [15]. Such guided principle and the conduct of professionalism are the foundation of the professional identify of the medical profession. Professional identify is what makes medical profession unique and what differentiates medical doctors from a ‘common individual’ and being a doctor. It is also what makes medical doctors perceive and conduct themselves as medical professionals [17]. Professional identity needs to be formed throughout medical training and further developed during practice [16].

International studies confirm that medical doctors are facing a ‘value-threatening’ problem and fading respect and trust from patients [18] with one of the root causes being the lack of professional identity demonstrated amongst the medical workforce [19]. Medical doctors’ professional identify is what differentiates them from a ‘common individual’ and being a doctor and refers to how they perceive and conduct themselves as medical professionals [17]. Without the formation of strong professional identity and the demonstration of high standards in both clinical and ethical aspects, acquiring all required medical knowledge and skills cannot guarantee success and confidence in medical practice [19]. Professional identify is initially developed and formed during the process of medical education when core elements of professionalism for medical practitioners is taught and reinforced in addition to learning science and medical practice and further evolved during internship and the early years of medical practice [20]. This learning process is an important component of competence-based education for medical doctors which involves the development of values, ethical principles and their performance standards [21]. It is also a change process when skills, knowledge and the values required for successful practice of medicine is combined with individual doctors’ own personal value and identity [22]. The development of core elements required for the demonstration of professionalism in medical practices and principle of putting patients’ welfare ahead of all other considerations.

However, traditional medical education has focused more on developing medical graduates’ technical stills and the understanding of science and best practice, rather than developing a good understanding of what professionalism entails for medical practice [22]. Even if professionalism has been taught, the end goal, the demonstration of the successful development of professional identity on which their professionalism in clinical practice and in working with patients and the general public, has not been achieved. The importance of assisting medical graduates in the formation of professional identify has started to receive recognition in medical schools in developed countries over the last 15 years and incorporated in the medical curriculum [23]. However, no evidence has been found that such a development has influenced the revolutionary changes in the medical curriculum in the medical schools and medical education in China. Moreover, it is believed that little training had been offered to developing medical graduates’ communication and interpersonal skills and their understanding of the social context of medicine [24]. The three principles of professionalism, i) the prioritization and welfare of patients; ii) patient autonomy, and iii) social equity may not be fully understood and used to guide clinical practice [25].

In order to develop a medical workforce that is successful in providing quality medical care to patients in the Chinese hospitals, an understanding of the extent that current medical doctors can demonstrate their medical professional identity in the process of diagnosis and treatment to patients is necessary. It will help identify the potential areas of improvement that can ultimately improve the doctor-patient relations in the long term, inform the revision and advancement of medical curricula for university programs, and the development of organisation-based strategies to improving the competence of medical graduates and practitioners. The focus of the paper is to provide evidence to answer the following question. How well can medical doctors working in Chinese public hospitals demonstrate their professional identify in their practice?

## Methods

Paper-based questionnaire including multiple choice questions were sent to doctors and patients. The surveys were conducted within 4 weeks in October 2019. One Level III, Qian FoShan hospital (QFSH), and one Level II, LaiWu hospital (LWH), located in Jinan, the capital city of Shandong Province, were selected. The first target population were medical doctors working in the medical inpatient units (*n* = 328) or the surgical inpatient units (*n* = 351). Eighty percent of all medical doctors from both hospitals who were working in the targeted units during the survey period were invited to participate in the study. The second target population were inpatients of the medical or surgical inpatient units at the time of receiving discharge notice. Table 1 indicates the actual inpatient target population.

**Table 1:** Target population – inpatients

Hospitals	Target population - Inpatients	Annual volume	Monthly turnover	Minimum sample size	% of total target population
QFSH	Medical Inpatient Units	55,561	4500	480	≈10%
	Surgical Inpatient Units	52,768	4400	480	≈10%
LWH	Medical Inpatient Units	6990	583	120	≈21%
	Surgical Inpatient Units	4769	397	120	≈30%

*QFSH* Qian FoShan hospital. *LWH* LaiWu hospital

For the patient paper-based survey, the questionnaires were completed either by the patients themselves, their family members or by the nurse who assisted them in the discharge process. Both surveys took approximately 15 min to complete. A participant information sheet together with informed consent was given together with the survey questionnaire to all participants.

### Questionnaires

Both questionnaires were developed in English before translation into Mandarin Chinese. To maintain accuracy, they were then back translated into English by an independent collaborator and any adjustments were applied to the Chinese version. The doctor questionnaire included 37 multiple choice questions. In addition to demographic, educational background and clinical position and ranking, the questionnaire also asked for doctors’ views on a number of responsibility areas in relation to professional practice in medicine as discussed in background section of the paper. The patient questionnaire included 38 multiple choice questions. In addition to demographic information, the questionnaire also included questions on past experience in receiving medical services which were closely related to the professional practice of their doctors which correlate to the questions included in the doctors’ survey. The patient survey served as a validation of the questions included in doctors survey.

In addition to some questions collecting data to understand the demographic and personal characteristics of participants such as education (doctors only), income (patients only), clinical position (doctors only), the main questions can be grouped into the four categories based on key areas of professional identify and professional practices to be demonstrated by medical doctors as discussed earlier [5, 6, 9, 10, 12, 15]

1. Patient consultation and consideration of patient preference
2. Professional and ethic conducts
3. Communication and trust
4. Roles and guiding principles of the medical profession

### Data analysis

Data from the two sets of the paper-based questionnaires were manually double entered into two MS Excel files and underwent error checking. The data were then imported into two IBM SPSS version 25 files. Descriptive statistics were performed on all variables separately by respondent. The dependent variables were then analysed by independent variables such as hospital, unit type, doctor seniority, age, gender by cross tabulation and chi square tests.

### Ethical considerations

The study received ethics approval from the Research Committee of Qianfoshan Hospital and LaiWu Hospital for conducting the study with their doctors and patients as described in the method section. The study also received ethics clearance from La Trobe University Ethics Committee. Special consideration was also given to patient involvement, only inviting inpatients after they have received the discharge notice. Both surveys did not collect personal and identifiable information to protect participants’ anonymous.

## Results

The study yielded high response rates of 90% for the doctors’ survey and 91% for the inpatient survey as detailed in Table 2. Table 2 details the actual number of questionnaires sent our during the study period and the questionnaires that were completed and returned.

**Table 2:** Target population and participation by hospital and unit

Target Unit	Hospital	Target population Doctors			Target population Inpatients		
		Total target	Total participants	Response rate	Total target	Total participants	Response rate
Surgical Units	QFSH	308	279	91%	480	413	86%
	LWH	43	37	86%	88	84	95%
Medical Units	QFSH	277	251	91%	553	539	98%
	LWH	51	47	92%	152	148	97%
Total		679	614	90%	1273	1184	93%

The seniority of the study doctors was as follows: chief physicians (19.4%), deputy chief physicians (20.1%), attending physicians (29.4%) and residents (31.1%).

**Table 3:** Percentage of response categories by respondent type and question

Questions	Strongly disagree	Disagree	Neutral	Agree	Strongly agree
**Doctors**					
Patients make better choices if involved in the treatment planning process	11.7	14.8	23.7	27.2	22.5
It is important to consult patients to achieve better treatment outcomes	1.8	6.7	10.5	40.9	40.1
It is unnecessary to consult patients before determining diagnostic procedures	44.8	19.6	13.6	12.6	9.3
**Patients**					
Doctor consulted you before diagnostic procedures	4.4	5.9	7.7	22.8	59.1
Doctor consulted you before deciding treatment plans	2.2	3.4	7.8	21.3	65.4

**Table 4:** Comparative analysis for group one questions

Questions	Comparative analyses
**Doctors**	
Patients make better choices if involved in the treatment planning process	Doctors in surgical units were significantly more likely to agree to this question than doctors in internal medical units. Chi square = 27.700, df = 4, *p* < 0.0001.
It is important to consult patients to achieve better treatment outcomes	Doctors in surgical units were significantly more likely to agree to this question than doctors in medical units. Chi square = 15.132, df = 4, *p* = 0.004.
**Patients**	
Doctor consulted you before diagnostic procedures	Patients at LWH were significantly more likely to agree to this question that patients at QFSH. Chi square = 33.807, df = 4, *p* < 0.0001. Patients in internal medical units were significantly more likely to agree to this question than patients in surgical units. Chi square = 18.026, df = 4, *p* = 0.001.
Doctor consulted you before deciding treatment plans	Patients at LWH were significantly more likely to agree to this question than patients at QFSH. Chi square = 23.954, df = 4, *p* < 0.0001.

Results of the questions from both surveys on doctors and patients are presented under four question groups. Two tables are provided under each group of the questions. One table presents details the percentage of doctors and patient responding to difference choices to various questions. Another table provides details of the comparative analysis performed looking into whether there are significant differences in the views between doctors and patients, surgical and internal medicine units and hospitals.

### Group one questions – consulting patients and considering their preferences (Tables 3 and 4)

Both surveys asked questions relating to the consultation process prior to determining types of diagnostic procedures and treatments. A 5-point Likert agreement scale was used. Overall, approximately 20% of doctors either disagreed or strongly disagreed that it is important to consult patients prior to determining the diagnostic procedure and treatment plan. The answers were similar with what was perceived by patients as 80–85% of them agreed that they were consulted prior. However, less than half of the doctors agreed or strongly agreed that patients made better choices if they are involved in the treatment planning process.

### Group two questions – professional and ethical conduct (Tables 5 and 6)

Doctors and patients were asked six questions in relation to doctors’ professional and ethical conduct using 5-Likert agreement or frequency scales.

**Table 5:** Percentage of response categories by respondent type and question

Questions	Strongly disagree	Disagree	Neutral	Agree	Strongly agree
**Doctors**					
There are enough rules to guide you when making decisions in the face of ethical dilemmas	1.1	2.3	7.9	23.1	65.6
Doctors should have the ability to make own decision without being influenced by external factors / interference	2.3	2.0	7.7	26.6	61.4
Doctors should have the ability to make decisions without being influenced by patients’ preferences	3.9	7.0	13.1	29.2	46.7
**Questions**	Never	Rarely	Sometimes	Often	Always
**Doctors**					
How often do you consider ethical implications in your clinical decision making?	0.3	0.3	1.8	20.5	77.1
How often do you prescribe tests and procedures that are not necessary to patients, but for generating profit for the department and/or hospital?	51.9	26.0	9.0	6.5	6.5
**Patients**					
Patients give doctors gifts because they want better treatment	47.5	12.1	14.0	8.4	18.0

**Table 6:** Comparative analysis for group two questions

Questions	Comparative analysis
**Doctors**	
How often do you prescribe tests and procedures that are not necessary to patients, but for generating profit for the department and/or hospital?	Doctors from internal medical units were significantly more likely to select the answers of rarely or never than those from surgical units. Chi square = 11.908, df = 4, *p* = 0.018.
**Patients**	
Patients give doctors gifts because they want better treatment	Patients from LWH were more likely to agree or strongly agree that the reason for them to give doctors presents were for better treatment than patients from QFSH. Chi square = 33.672, df = 4, *p* < 0.0001.

Around 90% of doctors indicated that there were enough rules to guide them when making decisions in the face of ethical dilemmas and agreed or strongly agreed that they should have the ability to make decisions without being influenced by external factors and interference. However, 76% of doctors agreed or strongly agreed that they should make decisions without being influenced by patients’ preferences. Only 77% of doctors confirmed that they always considered ethical implications when making clinical decisions. Twelve percent of them indicated that they often or always prescribed tests and procedures that are not necessary to patients, but for generating profit for the department. On the other hand, 26% of patients often or always give gifts to doctors because they wanted better treatment.

### Group three questions – communications and trust (Table 7)

Ten questions were asked of doctors or patients in relation to doctors’ professional and ethical conduct using the 5-Likert agreement or frequency scales.

**Table 7:** Comparative analysis for group two questions

Questions	Comparative analysis
**Doctors**	
In the past, you had concerns about your own safety when treating patients in critical condition	Doctors from QFSH were more likely to agree to this question than doctors at LWH. Chi square = 10.486, df = 4, *p* = 0.033 Doctors from surgical units were significantly more likely to agree with the question than those from internal medical units. Chi square = 12.652, df = 4, *p* = 0.013
**Patients**	
My experience has positively changed my perceptions of doctors**^**	Patients from LWH were more likely to have changed their respect for doctors than patients from QFSH. Chi square = 39.729, df = 4, *p* < 0.0001.

Less than 50% of doctors agreed or strongly agreed that patients demonstrated high level of respect to them. This contrasts to 92% of patients confirmed that they showed respect to their doctors during the diagnostic and treatment process. Around 76% of doctors and 81% of patients agreed or strongly agreed that doctors were able to commit adequate time on consultation with patients during the diagnostic and treatment process. Moreover, around 85% patients agreed or strongly agreed that doctors were able to explain complex process to them patiently and were able to use plain language to explain medical terminology when needed. The percentage is higher than the responses received from doctors when 96% of them indicated that they often or always devoted enough time on patient consultation. In addition, patients also asked whether they agreed that doctors asked them to accept unnecessary diagnostic test/procedures and the reasons behind the decision. Fifteen percent of patients answered ‘Yes’. Amongst them, 62% indicated the reason being ‘Doctors prescribe unnecessary test as a way of self-protection’, 26% chose the reason of ‘making profits for the hospitals, and another 12% chose the reason of ‘due to the inexperience of the doctors’.

Doctors were also asked to indicate the five key reasons for disputes between doctors and patients. ‘Mistrust from patients and their family members’ and ‘patients’ unrealistic expectation of treatment outcomes’ were the top two reasons which were agreed by 91 and 87% of doctors respectively.

### Group four questions – roles and guiding principles for the medical profession

Doctors were asked to indicate their guiding medically related philosophies by selecting at least one answer from a list of four choices:

1. to care for patient as a whole including his/her physical and psychological well-being
2. to preserve or restore patients’ physical health
3. to provide health education, promotion and prevention to patients and community
4. to provide services on demand

A majority of the participants selected more than one answer. All of the answers were selected by more than 75% of all participants, with 92% of participants agreeing with answer one and 87, 84, and 76% of participants agreed with answers 2, 3and 4 respectively.

Patients were also asked about their experiences of doctors in ‘care for patient as a whole including his/her physical and psychological well-being’ and ‘providing health education, promotion and prevention to patients and community’. Slightly more than 60% of the patients indicated that doctors always performed these roles.

## Discussion

Many factors have contributed to the tense doctor-patient relationship in the Chinese public hospital system. Studies have confirmed that mistrust by patients and families, poor communication by medical professionals and system level barriers being the major reasons [10, 11]. However, there are limited studies trying to understand the fundamental reasons why the reputation of doctors, usually a respected profession, has deteriorated. As discussed earlier, professional identify specific to the medical profession is what makes the vocation unique [15]. It is the authors’ view that regaining the respect and trust from patients and the public by reforming the professional identify amongst the profession is a key opportunity not to be ignored. Hence, an understanding of how well doctors in Chinese public hospitals can demonstrate their professional identify can contribute to the improvement of doctor-patient relationship in the long term. The study divided the three principles of professionalism and moral responsibilities of the profession into four key areas (as explained in the methods) with specific questions developed for doctors and patients to answer. With a high response rates from both doctors and patients, the study was able to answer the questions leading to meaningful suggestions made later in the paper.

Empirical evidence has confirmed patient participation contributes to quality of care and patient outcomes [26]. Patient consultation is key part of process of participation. However, its importance was only confirmed by 80% the doctors who recognised that patient consultation could lead to better patient outcomes. Only 50% of doctors believed that involving patients in the treatment planning process could assist patients with making better treatment choices. This is consistent with the fact that 20% of all doctors did not find consulting patients before determining diagnostic procedures necessary. The study also found that doctors from surgical units were more likely to consult patients than doctors in the internal medicine units which might reflect the standards of surgical procedures when patient consent is required prior to performing the procedure. However, recognising the importance and benefits of patient consultation is critical to putting patients’ preferences and well-being ahead of other consideration – a key aspects of medical professional conduct [18, 27].

Maintaining ethical principles is a core element of medical practice. Despite more than 90% of doctors confirmed that there were enough rules to guide them when making decisions in the face of ethical dilemmas, less than 80% of them often considered ethical implications when making clinical decisions for patients and had the ability to make decisions without being influenced by external factors or interference. In addition, 76% of doctors agreed that they should make decisions without being influenced by patients’ preferences which contradicts one of the principles of professional practice in medicine – putting patients’ preferences in all consideration [15, 18]. The inconsistency in practice amongst the medical workforce in the same hospital and the lack of consideration of patients’ preferences in diagnostic and treatment process has no doubt violated the trust from patients and families.

The fact that 12% of doctors often prescribed tests and procedures that were not necessary to patients, but for generating profit for the department, 26% of patients often gave gifts to doctors for the purpose of getting better treatment, and two thirds of patients believed that doctors prescribed unnecessary diagnostic procedure for ‘self-protection’ further confirms the existence of mistrust that patients have in doctors and also doctors have in patients. Furthermore, the study also found that only 76% of doctors felt that they had the respect from patients and more than 60% of them were concerned about their own safety when treating patients with critical conditions, again confirming the tense relationship between doctors and patients and the fear that large numbers of doctors are working under. As mentioned in the literature, this will inevitably turn doctors into a defensive mood, further exacerbating the already challenged doctor-patient relationship [12, 13]. Strategies are required to addressing these issues urgently to avoid further damaging the already tense doctor-patient relationships.

A encouraging result was that some aspects of doctors’ professionalism were confirmed by patients: i) that doctors were able to commit adequate time for patient consultation, and ii) had the ability in using plain language to explain complex treatment processes and medical terminology. The vast majority of doctors understood their social roles in caring for patient as a whole including their physical and psychological well-being and preserving or restoring patients’ physical health. This is something that should be recognised and encouraged.

The study implies that the further revision of medical curriculum is necessary in China. Medical education has been through significant change as a result of the Chinese Higher Education Reform, with the integration of medical colleges and comprehensive universities [28] and the implementation of a standardized three-year graduate medical education program at 559 nationally distributed commissioned teaching hospitals as two key foci. Such changes are aimed at meeting international standards and aligning with the established clinical competency requirements [29]. To achieve the aim of improving healthcare quality of the new national policy - Healthy China 2030 - a large number of well-trained medical professionals are required. From the authors’ views, one of the pressing issues is the formation of a strong professional identity regaining the respect and trust from the public, not only improving the doctor-patient relationship and patient satisfaction (a key indicator of quality of healthcare services), but also reducing the burn out and turnover amongst the medical workforce.

### Study limitations

The main limitation of the study was the lack of a direct link between the two study populations, doctors and patients apart from the common feature of the hospitals and departments. In addition, there were some mismatches between the questions posed to the two populations.

## Conclusions

Implications for practice and policy development.

A reduction of medical disputes and the improvement of doctor-patient relationships requires systematic change over the long-term. Firstly, a review and improvement of medical curricula is required to teach medical graduates professionalism and assist them to develop their professional identify, focusing their clinical practice around the patient as a whole person and delivering the best service outcomes. Secondly, investment in professional development and support in the early years of clinical practice of medical graduates will further develop and consolidate their professional identify. Thirdly, developing an organisation-based performance management framework together with implementation and evaluation strategies that can incentivise the demonstration of professionalism in clinical practice linking to patient satisfaction and the quality of service provision including desirable patient outcomes. Lastly, a health system reform agenda needs to take into consideration the negative impact of current funding models applied to public hospital systems in order to encourage the model of ‘pay for performance’ rather than ‘pay for volume’. This will ultimately improve the doctor-patient relationships, quality of service provision and clinical outcomes, and discourage distorted ‘defensive medicine’ practices.

## Supplementary Information

**Additional file 1.** Doctors Survey Chinese_English Version

**Additional file 2.** Patients Survey Chinese_English Version

## Data Availability

The datasets used and/or analysed during the current study are available from the corresponding author on reasonable request.

## Abbreviations

SARS: Severe acute respiratory syndrome
QFSH: Qian FoShan hospital
LWH: LaiWu hospital
