# Supplementary material for: Doctors in Chinese public hospitals: demonstration of their professional identities
Source: BMC Med Educ. 2020 Dec 10;20:501. doi: 10.1186/s12909-020-02339-3 (PMC7725881; doi:10.1186/s12909-020-02339-3)
Supplement: Supplementary file 1 — Additional file 1. Doctors Survey Chinese_English Version [file 12909_2020_2339_MOESM1_ESM.pdf]

Doctors' survey
医生调查问卷

1. Please select the classified level of the hospital where you are currently employed
   您现在就职医院的等级。
   1. 三级医院
   2. 二级医院
   3. 一级医院

2．Please select the department that you are currently working in
您目前就职的科室
   1. 内科系统
   2. 外科系统

3．What is the level of seniority of your medical position?
您目前的职称
   1) 主任医师 (Chief Physician)
   2) 副主任医师 (Associate Chief Physician)
   3) 主治医师 (Attending Physician)
   4) 住院医生 (Resident)

4. Have many years have you been working as a clinician in the hospital system (including hospitals that you previous worked currently working for)?
您做医院临床医生的年限？（包括以前和现在工作的医院）

5. How many years have you been employed by the current hospital?
您在目前就职医院的年限？

6. What is your age? 您的年龄

7. What is your gender?
   1) Male
   2) Female

您的性别
   1) 男
   2) 女

8. Do you have any of the following degrees?
   1) TAFE
   2) Bachelors
   3) Master
   4) Doctorate or PhD

您拥有的所有学位 (可以多选)
   1) 专科文凭
   2) 学士学位
   3) 硕士学位
   4) 博士学位

9. Please select the discipline(s) of all of the degrees that you obtained

1) Medicine
2) Nursing
3) Management
4) Others (please key in details)

选择您各个学位对应的所有专业（可以多选）

1) 医学
2) 护理
3) 管理
4) 其他 __________

All of the below questions will be provided the same 5-point Likert Scale for selection of answer

1) Strongly disagree
2) Disagree
3) Either agree or disagree
4) Agree
5) Strongly agree

请根据个人情况对以下问题作出选择

1) 完全不同意 (代表同意程度约 0%)
2) 小部分同意（代表同意程度约 25%）
3) 中立 （代表同意程度约 50%）
4) 大部分同意 （代表同意程度约 75%）
5) 完全同意（代表同意程度约 100%）

10.From your experience, patients' improved knowledge of medicine has pressured doctors' to be more rigorous in diagnosis and treatment?

根据您的经历，患者医学知识的增加需要医生更加严谨细致。

11. From your experience, patient can receive better treatment outcome if they are involved in the treatment planning process?

根据您的经历，如果让患者参与制定治疗方案，会提高治疗效果。

12. From your experience, it is important for patients to be involved and consulted during the diagnosis and treatment process in order to receiving better treatment outcomes?

根据您的经历，为了达到更好的治疗结果，在诊疗过程中参考和征询患者的意见，是很重要的。

13. From your experience, patients are not capable of making rational decisions regarding their health care

根据您的经历，患者对自己的治疗方案及保健服务并不能理性地做出选择。

14. From your experience, it is not necessary to consult patients the type of assisted diagnostic procedure

根据您的经历，没有必要与患者商量必须进行哪种辅助诊断。

15. You are able to devote adequate time to each of your patient during the diagnostic and treatment process

在诊疗过程中，您能够对每一位被诊治的患者投入足够的时间。

16. There are sufficient rules to guide you when making decisions in the face of ethical dilemmas

当诊疗行为需要考虑道德规范问题时，您有明确的准则依据。

17. Doctors should make their own judgement rather than relying on rules
医生应该具有排除各种社会因素干扰的情况下，独立作出判断的能力。

18. Doctors should make their own judgement without concerning patients' preference
医生应该在不受患者意愿干预的情况下，做出自己的判断

19. From your experience, patients demonstrate high level of respect to their doctors
根据您的经历，患者会充分尊重医生

20. In the past, you had concerns about your own safety when treating patients in critical condition
以往治疗急危重症患者的时候，您担心过自己的个人安全

21. Did you feel well supported by your superior or hospital when there were disputes between you and your patients
根据您的经历或者跟您紧密合作同事的经验，当发生医患纠纷时，您或您的同事能够得到上级医生或医院管理体系的理解与支持

22. Do you have confidence that you will be well supported by your superior or hospital when there are disputes between you and your patients?
当发生医患纠纷时，您或您的同事相信能够得到上级医生或者医院管理体系的理解与支持

All of the below questions will be provided the same 5-point Likert Scale for selection of answer

1) Never
2) Rarely
3) Sometimes
4) Often
5) Always

请根据个人情况对以下问题作出选择

1) 从来不会（代表出现频率约 0%）
2) 极少（代表出现频率约 25%）
3) 偶尔（代表出现频率约 50%）
4) 经常（代表出现频率约 75%）
5) 总会如此（代表出现频率约 100%）

23. How often do you consult patients before determining the types of test and procedures to be performed that can assist with making diagnosis decision?
您在决定必需的检查检验方案时，会征求患者的意见吗

24. How often do you consider ethical implication in your clinical decision making?
在作出医疗决策时，您会考虑道德规范吗？

25. How often do you need to use plain language to explain diagnostic and treatment procedure to patients rather than using medical terminology?
在向患者解释诊疗问题的过程中，您会用通俗语言取代医学术语吗？

26. How often are you able to reduce resistance from patients when asking questions that are confidential and private in nature?

当您询问患者隐私问题时，您能用适当的方法减弱患者的抵触情绪吗？

27. How often do you prescribe tests and procedures that are not necessary to patients, but for generating profit for the department and/or hospital?
您会因为所在部门或医院创收而要求患者做不必要的检查吗？

All of the below questions will be provided the same 5-point Likert Scale for selection of answer

1) Never
2) Rarely
3) Sometimes
4) Often
5) Always

请根据个人情况对以下问题((31-33)作出选择

1) 从来没有（代表出现频率约 0%）
2) 极少 （代表出现频率约 25%）
3) 偶尔 （代表出现频率约 50%）
4) 经常 （代表出现频率约 75%）
5) 总是 （代表出现频率约 100%）

28. How often do you face with ethical dilemmas in your work that are hard to resolve?
您在工作中遇到过难以解决的与道德规范有关的难题吗？

29. How often do you encounter patients recording the diagnostic and treatment process by audio or video without your consent?
您是否遇到过在没有经过您同意的情况下，患者对诊疗过程进行录音或者录像的情形？

30. How often do you terminate the treatment if you encounter the above situation as in question 29?
如果遇到患者对诊疗过程进行录音或者录像的情形，您会中断治疗吗？

31. How often were you able to complete the examination process when you encountered resistance from patients when performing physical examination on private body part?
当您检查患者隐私部位遇到抗拒时，您能顺利完成检查吗？

Multiple choice, one answer only 请在下面问题中，选出一个最合适的答案。

32. The guiding philosophy of your being a doctor is
1) to care for patient as a person including his/her physical and psychological well-being
2) to preserve or restore patients’ physical health
3) to serve the community
4) to provide services on demand

作为一名医生，您对自己的职业界定是：
1) 对每一位患者都一视同仁，关心他们的身心健康
2) 帮助患者恢复并保持健康
3) 有对群众传授预防疾病、维护健康知识的责任

4) 当群众有需要的时候，就会提供医疗健康服务

33. Do you agree with which of the following statement in relation to patients' consent on tests?
   1) patients' consent is needed in all procedures, tests and treatments
   2) patients' consent is needed in major procedures, tests and treatment
   3) patients' consent is needed in non-standard procedures, tests and treatment only
   4) Patients' consents to procedures, tests and treatments are only required when patient is required to make the decision
   5) no patients' consent to procedure, tests and treatment is necessary as doctors should always have the final say

   决定检查项目时，对事先征求患者同意，您赞同以下哪一个做法？
   1) 所有检查都需要事先获得患者的同意
   2) 主要的检查，需要事先获得患者的同意
   3) 只有非常规性检查，才需要事先获得患者的同意
   4) 只有必须患者同意才能进行的检查，才需要事先征得患者的同意
   5) 任何情况下都无需获得患者的同意，因为医生有最终决定权

34. Do you agree with which of the following statement in relation to patients' consent on treatment?

1) patients' consent is needed in all procedures, tests and treatments
2) patients' consent is needed in major procedures, tests and treatment
3) patients' consent is needed in non-standard procedures, tests and treatment only
4) Patients' consents to procedures, tests and treatments are only required when patient is required to make the decision
5) no patients' consent to procedure, tests and treatment is necessary as doctors should always have the final say

决定治疗方案时，对事先征求患者同意，您赞同以下哪一个做法？
1) 所有检查都需要事先获得患者的同意
2) 主要的治疗，需要事先获得患者的同意
3) 只有非常规性治疗，才需要事先获得患者的同意
4) 只有必须患者同意才能进行的治疗，才需要事先征得患者的同意
5) 任何情况下都无需获得患者的同意，因为医生有最终决定权

35. With whom do you think it is appropriate to discuss a patient's case first when patients are in critical condition?
   1) Patients' themselves
   2) Immediate family members
   3) Patient's relatives
   4) Other doctors
   5) Superior
   6) Others

当患者病情重危时，您认为第一时间内的告知人是谁？
   1) 患者本人
   2) 患者的直系亲属
   3) 患者的亲戚（非直系亲属）
   4) 上级医生或上司
   5) 其他医生
   6) 其他人

36. How many of your patients can you recognise by face?
   1) More than 75%
   2) 50 – 74%
   3) 25-49%
   4) Less than 25%
   5) None

您能够认出多少您曾经的患者?
   1) 超过 75%的患者
   2) 50-74%的患者
   3) 25-49%的患者
   4) 少于 25%的患者
   5) 一个也认不出

37. From your experience, what are the primary reasons that lead to dispute between doctors and patients
   1) Patients and/or carers' mistrust
   2) Patients unrealistic expectation of treatment outcomes and prognosis
   3) Patients experienced complication as a result of lack of compliance to medical order
   4) Complexity of hospital procedure and protocol leading to the inconvenience to patients and/or carers
   5) Necessary treatment and procedures are unaffordable to patients
   6) Long waiting for consultation/procedure/treatment
   7) Patients suffered from preventable adverse outcomes
   8) Patients suffered from unavoidable adverse outcomes under the current medical knowledge and treatment capacity
   9) Ineffective communication between patients and doctors

   根据您的经历或观察，您认为造成医患纠纷的主要原因是（限选 5 项）
   1) 患者或患者亲属的不信任
   2) 患者对疗效和预后抱有不切实际的期望
   3) 患者不遵医嘱导致出现不良结果
   4) 医院流程与操作的复杂性给患者们带来不便
   5) 患者难以负担诊疗必需的费用
   6) 诊疗的等待时间过长
   7) 医疗过失
   8) 当今医学水平所限导致患者出现不良结果
   9) 医患沟通不顺畅
