# Supplementary material for: Doctors in Chinese public hospitals: demonstration of their professional identities
Source: BMC Med Educ. 2020 Dec 10;20:501. doi: 10.1186/s12909-020-02339-3 (PMC7725881; doi:10.1186/s12909-020-02339-3)
Supplement: Supplementary file 2 — Additional file 2. Patients Survey Chinese_English Version [file 12909_2020_2339_MOESM2_ESM.pdf]

医生职业特征调查问卷（患者版）

填写日期:　　2019 年　　月　　日

填写时间:　　上午〇

下午〇

问卷填写人:　　患者本人〇

患者家属〇

医护人员〇

问卷编号: ____________________

所在科室:　　内科系统〇外科系统〇

（由项目成员数据输入时填写）

2019 年 9-11 月

山东第一医科大学第一附属医院（山东省千佛山医院）

医生职业特征调查问卷（患者版）

填写日期:　　　　2019 年　　月　　日

填写时间:　　　　上午〇

　　　　　　　　下午〇

问卷填写人:　　　患者本人〇

　　　　　　　　患者家属〇

　　　　　　　　医护人员〇

问卷编号: ____________________

所在科室:　　　内科系统〇外科系统〇

（由项目成员数据输入时填写）

2019 年 9-11 月

山东第一医科大学第一附属医院（山东省千佛山医院）

La Trobe University

参与研究同意书

我已经阅读并了解整个研究的相关内容。在回答问卷前，问卷调查员给予我提问的机会，我提出的所有问题均已得到解答。我知道该项调查是完全自愿与义务性的。可以在任何时候退出问卷调查。

当我开始填写问卷时，代表我

1. 同意参与该项调查
2. 同意我提供在问卷里的信息可以用于撰写论文、演讲或学术期刊文章。而且我知道我的个人信息不会因此被泄露。

希望我提供的信息能够对该研究或日后的相关研究有所帮助。

一、 选择题
（除第 8 题外，请只选择一个答案）

1、性别

〇男

〇女

2、居住地

〇农村

〇乡镇

〇城市(县城及以上)

3、年龄

〇17 岁及以下

〇18 岁－34 岁

〇35 岁-54 岁

〇55 岁－59 岁

〇60 岁及以上

4、教育程度

〇小学及以下

〇初中

〇高中

〇本科/大专

〇研究生及以上

5、您目前或退休前的工作性质是

〇医务人员

〇非医务人员

〇无业者

6、选择医生的方式

〇网上查询

〇亲友推荐

〇随机挂号

〇曾经就诊或熟悉的医生

7、您个人税前年收入(元)

〇1 万以下

〇1 万-5 万

〇5 万以上

8、诊疗过程中，影响您对诊疗服务满意度的主要因素（最多选择 3 项）

〇就诊硬件环境

〇医疗效果

〇医护人员态度

〇医护人员仪容

〇就医流程便利程度

〇诊疗时间的长短

9、在过去的诊疗经历中，您是否觉得医生要求做的辅助检查是不必要的。

〇是

〇否

10、您认为医生要求做不必要的辅助检查的原因是

〇医生经验不足

〇医院经营目标要求

〇医生自我保护意识（虽然他们经验足够）

11、如果您或亲朋好友有过重病经历时，医生是如何履行病情告知义务的？

〇医务人员必须如实告知病人病情

〇医务人员暂不告知病人，而是先与患者亲属商量

〇医务人员根据实际情况处理

〇没有这种经历

12、根据您或亲朋好友的经历，当发生医患纠纷时，首先介入调查的是哪一项？

〇政府卫生主管部门

〇医疗行业协会

〇所在医院

〇媒体报道

〇没有这种经历

二　量表选择题

题 13–26，　请您根据‘5 级量表’做出选择，如实反映过去就诊经历的实际情况

1 从来没有　(代表出现频率约 0%)

2 极少　（代表出现频率约 25%）

3 中立　（代表出现频率约 50%）

4 经常　(代表出现频率约 75%)

5 总是　(代表出现频率约 100%)

	问题	1	2	3	4	5
13	过去就诊过程中，医生决定检查检验时，征求过您的意见。	1	2	3	4	5
14	过去就诊过程中，医生决定治疗方案时，征求过您的意见。	1	2	3	4	5
15	过去就诊过程中，医生赢得了您信任。	1	2	3	4	5
16	过去就诊过程中，医生询问与疾病相关的隐私问题让您反感。	1	2	3	4	5

17	过去就诊过程中，医生检查与疾病相关的隐私部位让您反感。					
18	过去就诊过程中，医生都会用足够的时间询问病情。	1	2	3	4	5
19	过去就诊过程中，医生要求做了不必要的检查检验。	1	2	3	4	5
20	过去就诊过程中，医生会耐心解释繁杂的诊疗过程。	1	2	3	4	5
21	过去就诊过程中,医生对每一位患者都同样关心、照顾。	1	2	3	4	5
22	过去就诊过程中，医生都会传授预防疾病、维护健康的知识	1	2	3	4	5
23	医生介绍病情时,经常使用难以明白的医学术语。	1	2	3	4	5
24	过去就诊过程中，当遇到不明白的医学术语时，医生都能用通俗易懂语言解释清楚。	1	2	3	4	5
25	过去的诊疗过程中，我对医生是尊重的。	1	2	3	4	5
26	根据您或亲朋好友的经历，医生会事先解释诊疗过程中病情恶化的可能性	1	2	3	4	5

题 27-35， 请您根据‘5 级量表’做出选择，如实反映过去就诊经历的情况

1 完全不同意 (代表同意程度约 0%)
2 少部分同意 (代表同意程度约 25%)
3 中立 (代表同意程度约 50%)
4 大部分同意 (代表同意程度约 75%)
5 完全同意 (代表同意程度约 100%)

27	医生是高风险的职业。	1	2	3	4	5
28	医生是工作量繁重的职业。	1	2	3	4	5
29	医生是培养时间长、技术含量高的职业。	1	2	3	4	5
30	医生是高收入阶层。	1	2	3	4	5
31	患者有权在诊疗过程进行录音、录像。	1	2	3	4	5
32	患者给医生送礼是为了得到更好的诊疗。	1	2	3	4	5
33	过去的医疗经历让我对医生的看法变好。	1	2	3	4	5
34	现在患者医学知识的提高促使医生更加严谨细致。	1	2	3	4	5
35	当出现医患纠纷时，医院能客观地调查解决。	1	2	3	4	5

问卷到此结束，谢谢您的参与！请把问卷放进问卷收集箱里

Patient's questionnaire

A. Multiple Choice Questions (Please choose only one answer for each question except Question 8)

1. Gender
- ○ Male
- ○ Female

2. Residence
- ○ Rural area
- ○ Regional area
- ○ Metropolitan

3. Age
- ○ 17 years old or below
- ○ 18-34 years old
- ○ 35-54 years old
- ○ 55-59 years old
- ○ 60 years old or above

4. Education
- ○ Primary school or below
- ○ Secondary school
- ○ Senior secondary school
- ○ Bachelor/TAFE
- ○ Master or above

5. Occupation: current or before retirement
- ○ Clinical position
- ○ Non-clinical position
- ○ Unemployed

6. The way to choose doctors
- ○ Online searching
- ○ Recommendation from family or friends
- ○ Random booking
- ○ Previous or familiar doctors

7. Your annual income before tax
- ○ Below 10,000 RMB
- ○ 10,000-50,000 RMB
- ○ Above 50,000 RMB

8. During the treatment process, what are the main factors that could affect your satisfaction with the medical service? (You can maximally choose three options)
- ○ Hospital environment
- ○ Medical treatment effect
- ○ Medical staff attitude
- ○ Medical staff appearance
- ○ Convenience of the treatment process
- ○ Duration of the diagnosis and treatment process

9. In your previous experience, did you think the assisted tests required by the doctors was unnecessary?
- ○ Yes
- ○ No

10. In your opinion, what is the reason that doctors require patients to do the unnecessary assisted tests?
- ○ Doctors do not have enough experience
- ○ Generating profit for the hospital

○ Doctors' self-protect act (even when with adequate experience)

11. If you or your family were ever in critical condition, how did the doctor inform patients of the condition?

○ Medical staff inform patients of the actual condition
○ Medical staff discussed the condition with patients' family first without informing the patients
○ Medical staff will decide how to act on case to case basis
○ I don't have this kind of experience

12. According to your or your family experience, when there was dispute between a doctor-patient, which of the following should take place first?

○ Government health authority
○ Medical industry association
○ Hospital
○ Media report
○ I don't have this kind of experience

B. 5-point Likert Scale Questions

Based on your past diagnosis and treatment experience, use the 5-point Likert Scale to complete Question 13-26.

1 Never
2 Rarely
3 Sometimes
4 Often
5 Always

	**Question**	**1**	**2**	**3**	**4**	**5**
13	In the previous diagnostic and treatment process, doctors sought my permission before deciding to perform medical check up and test	1	2	3	4	5
14	In the previous diagnostic and treatment process, doctors sought my permission before deciding the medical treatment plan.	1	2	3	4	5
15	In the previous diagnostic and treatment process, doctors earned my trust.	1	2	3	4	5
16	In the previous diagnostic and treatment process, I dislikeded that doctors asked me questions related to the illness that are private and confidential	1	2	3	4	5
17	In the previous diagnostic and treatment process, I disliked when the doctor examined my private body parts that are					

	relevant to the illness.					
18	In the previous diagnostic and treatment process, doctors spent enough time asking me about the condition.	1	2	3	4	5
19	In the previous diagnostic and treatment process, doctors asked me to go through unnecessary examination and tests	1	2	3	4	5
20	In the previous diagnostic and treatment process, doctors patiently explained the complex diagnosis and treatment process to me.	1	2	3	4	5
21	In the previous diagnostic and treatment process, doctors showed the same level of care to each of their patients	1	2	3	4	5
22	In the previous diagnostic and treatment process, doctors taught me how to maintain health and prevent disease.	1	2	3	4	5
23	Doctors often use medical terms which are hard to understand to explain the condition to me	1	2	3	4	5
24	In the previous diagnostic and treatment process, doctors were able to use simple language to explain medical terminologies that I could not understand	1	2	3	4	5
25	In the previous diagnostic and treatment process, I showed doctors my respect	1	2	3	4	5
26	Based on my own experience or experience of my family members, doctors explained the possibility of adverse outcome/prognosis that we may encountered during the diagnosis and treatment process in advance	1	2	3	4	5

Based on your past diagnosis and treatment experience, use the 5-point Likert Scale to complete Question 27-35.

1 Strongly disagree
2 Disagree
3 Either agree or disagree
4 Agree
5 Strongly agree

27	Doctor is a high-risk profession	1	2	3	4	5
28	Doctors have heavy workload that is very complex	1	2	3	4	5
29	Doctor is a profession with high level skill requirements which take a long process to develop	1	2	3	4	5
30	Doctors' income are high	1	2	3	4	5
31	Patients have the right to record the diagnostic and treatment process.	1	2	3	4	5
32	The reason for patients give gifts to doctors gifts is to receive better diagnosis and medical treatment.	1	2	3	4	5

33	My previous experiences have positively changed my regards to doctors	1	2	3	4	5
34	The improved medical knowledge of patients has pressured doctors act more rigorous.	1	2	3	4	5
35	When a doctor-patient dispute occurs, the hospital can investigate and resolve the issues objectively.	1	2	3	4	5

问卷到此结束，谢谢您的参与！请把问卷放进问卷收集箱里

This is the end of the questionnaire. Thank you for your participation. Please put the questionnaire into collection box.
